# On the perspective of doctors’ intention—a hybrid BYOD model

**DOI:** 10.1186/s12913-025-12801-x

**Published:** 2025-10-14

**Authors:** Saima Nisar, Wan Rozaini Bt Sheik Osman, Alawiyah Bt Abd Wahab, Muhammad Shahzad Aslam

**Affiliations:** 1 School of Computing, College of Arts and Science, Universiti Utara Malaysia, Sintok, Malaysia; 2 PeerMetric Research Consortium, School of Traditional Chinese Medicine, Xiamen University Malaysia, Jalan Sunsuria, Bandar Sunsuria, Sepang, 43900 Selangor Malaysia; 3 School of Economics and Management, Xiamen University Malaysia, Sepang, Malaysia

**Keywords:** BYOD adoption model, Unified theory of acceptance and use of technology, Protection motivation theory, Healthcare professionals adoption intention, BYOD policies, Pakistan

## Abstract

**Background:**

Mobile devices enable doctors to make informed decisions that provide quality healthcare outcomes. With Bring Your Own Device (BYOD), privacy and security are considered primary concerns to doctors since they handle sensitive and highly confidential data.

**Objective:**

The study aims to identify the determinant factors that affect the adoption of BYOD among Pakistani doctors.

**Methods:**

A theoretical study was conducted to identify the determinant factors, and then a hybrid model based on the identified factors was proposed. The hybrid model was developed by integrating the UTAUT model and PMT. Performance expectancy, effort expectancy, social influence and facilitating conditions from the UTAUT model were incorporated with perceived vulnerability, perceived severity, response cost and self-efficacy factors from PMT. Data collection was performed based on snowball sampling and was realised through an online survey instrument on Facebook. A total of 245 licensed doctors from Pakistan participated in the survey.

**Results:**

The survey data were analysed using Structural Equation Modelling (SEM). The findings indicate that the hybrid model is an acceptable fit model as it explains 87 percent of the variance in behavioural intention. The results show that the UTAUT's performance expectancy, effort expectancy, facilitating conditions, and PMT's self-efficacy positively affected doctors' BYOD adoption, while PMT's perceived vulnerability and perceived severity had a negative impact.

**Conclusions:**

Theoretically, this study contributes to enhance the BYOD adoption model for the medical domain. The findings of this study also serve as a basis for hospital management to formulate and implement policies regarding data protection and security measures.

**Supplementary Information:**

The online version contains supplementary material available at 10.1186/s12913-025-12801-x.

## Introduction

In today’s fast-paced digital economy, every sector increasingly adopting digital transformation to stay competitive and improve operational efficiency [39]. Many countries have rapidly progressed in infrastructures, support mechanisms, and aligning Information and Communication Technologies (ICT) policy with healthcare vision [37, 78]. These technological advantages have sparked the attention of many countries to digitize healthcare and the increasing penetration of mobile devices have influenced their use in medical education and healthcare delivery [56]. Innovative technologies like mobile devices offer methods to enhance healthcare data access in Low and Middle-Income Countries (LMICs). Mobile devices are thought to help hospital staff work more efficiently, leading to better patient care. Since the Covid-19 pandemic, the use of remote and virtual healthcare services like telehealth and remote monitoring has increased significantly. Bring Your Own Device (BYOD) supports this by allowing healthcare providers to move around easily and coordinate patient care more effectively [109].

Bring-your-own-device (BYOD) is generally conceptualized as employees’ use of personal mobile devices to complete work-related tasks [55]. Driven by rapid advances in ICT and a recent increase in consumer ICTs in the workplace, BYOD is considered a form of Information Technology (IT) consumerization [109]. BYOD is already a working fashion, as the use of portable electronic devices has progressively risen in past years. BYOD applies to workers bringing their devices to the office [23].

Furthermore, previous studies find out that the acceptance of BYOD depends on the combination of related variables that must be synchronously taken into account and it depends on the individual qualities that need to be discussed [25, 81, 91]. The importance of variables that affects the acceptance of individuals may not be the same when studied within developed and emerging nations as the beliefs, morals and traditions of these countries are also unique, and this may influence their attitudes towards technology acceptance [1, 6, 8, 13, 17].

Despite the growing adoption of BYOD in healthcare, and extensive research exists on various perspectives of BYOD adoption, there remains a significant gap in research exploring doctors' perspectives, particularly their willingness to adopt BYOD, the concerns they face, and their firsthand experiences. Most of the studies concentrated on organisational risks such as virus attacks, employees' improper use of assets and information [114] and growing concerns for organisation information security management [89, 108]. Unauthorised employees' access to organisation information, download risky mobile applications and stolen; lost device [22] neglect safety issues about possible breaches of sensitive or secret details [108].

While numerous studies have explored BYOD in healthcare, the majority have focused on socio-technical aspects and its impact on clinical and administrative work [109], investigation of the role of BYOD devices in fostering cybersecurity awareness and its correlation with the productivity of healthcare professionals [67] and investigate security risk perception and safeguard adoption of mobile devices among medical practitioners and IT administrators [9]. Also, BYOD challenges and risks while managing and controlling corporate data and networks [12] and BYOD challenges when employees fail to comply with security policies [76]. Despite the acknowledged benefits of BYOD and its initiatives in recent literature, its adoption remains insignificant among doctors of developing countries, including Pakistan [58].

However, they neglect the perspective of the primary user—the doctors—who is expected to voluntarily use their personal devices for professional purposes. Without addressing the willingness of doctors to adopt BYOD, the effort to create a seamless BYOD environment becomes futile, as the success of this model hinges on their active and voluntary participation [22, 108, 110, 116]. This gap highlights the need to investigate doctors' intention to ensure BYOD implementation aligns with their needs and preferences, ultimately enhancing its effectiveness in clinical practice.

Doctors' intention to adopt BYOD is critical because they are the ones bringing their own devices into the professional setting [42]. Their concerns, motivations, and preferences are pivotal to the model's success, yet remain underexplored in the literature. Key questions arise about their privacy concerns when blending personal and professional data on a single device [64, 70]. This lack of clarity on privacy-related apprehensions, especially when the device is owned and controlled by the doctor, creates a significant barrier to understanding their willingness to adopt BYOD [22].

Privacy and security are primary concerns in healthcare communication technologies. These problems emerge as mobile phones handle sensitive data and highly confidential data. The confidentiality vulnerability from practitioners is an issue that needs to be considered so that only permitted people can access sensitive information. However, some studies have investigated information security-related empirical research. The increase of different devices has led to an increased risk of exposure to viruses, malware, and a host of other security issues and the potential leaking of sensitive material and data [14]. The challenge in adopting BYOD is ensuring the safety and security of the doctors' data and devices. Furthermore, using their own devices may instill greater confidence among doctors compared to using company-provided devices, which often require additional training or adjustment. Doctors may find personal devices more convenient, familiar, and efficient for professional use. Additionally, the cost of the device falls on the doctor, further emphasizing the need to understand their willingness and readiness to embrace BYOD.

The limitations of BYOD adoption are linked to several variables, representing the individual's characteristics and features of the technology itself. The importance of variables that affect individuals' acceptance may not be the same when studied within developed and emerging nations, as these countries' beliefs, morals, and traditions are unique. Pakistan's doctors represent the primary users of BYOD, and their acceptance of it can significantly influence its adoption. The study manifests a need for empirical evidence to identify and evaluate the determinant factors underlying how doctors in emerging nations might see and intend to adopt BYOD.

By addressing these gaps, this study seeks to contribute to a deeper understanding of doctors' intentions and motivations, which are crucial for the effective implementation of BYOD in healthcare settings specifically doctors' intention to accept health technologies to allow effective healthcare administration in Pakistan. Hence, a specific model is required to examine BYOD's intention among the doctors in Pakistan.

### Research model

**Fig. 1:**
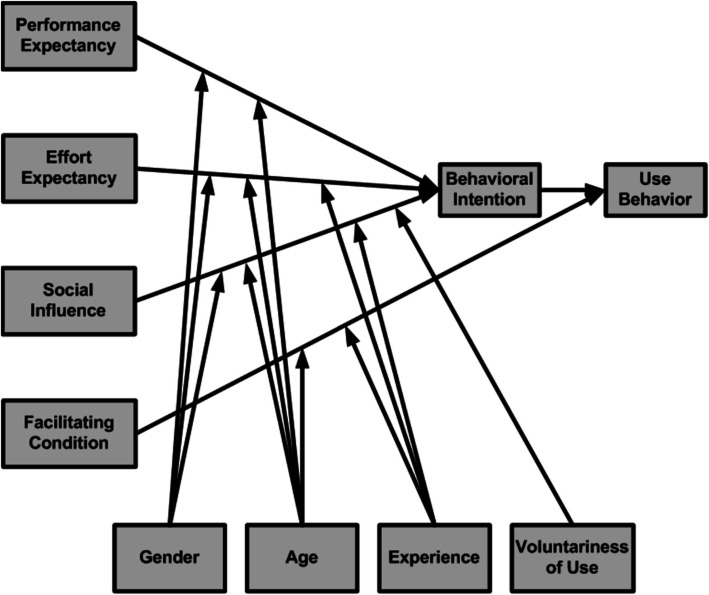
Unified Theory of Acceptance and Use of Technology (UTAUT) [101]

Venkatesh [101] presented Unified Theory of Acceptance and Use of Technology (UTAUT) in 2003 after reviewing eight models and theories, this model is suitable for defining employees' technology acceptance and use. UTAUT posits several factors that shape an individual's inclination to adopt a technology [2]. The model presented three variables: performance expectancy, effort expectancy, and social influence that directly affect behavioural intention. Facilitating conditions and behavioural intention direct affect technology use. Those relationships moderated by age, gender, experience and voluntariness [101], as displayed in Fig. 1.

Nevertheless, in a healthcare context, the UTAUT model does not seem to have a similar effect as in any other settings in order to describe doctors' attitudes, and the variance described in previous studies seems to be noticeably smaller than the variance defined in the UTAUT model, reflecting a limitation in the healthcare system [1, 15, 19, 52, 103]. These research results supported this study to integrate the UTAUT model with other factors to measure its significance in the Pakistani doctors' context.

**Fig. 2:**
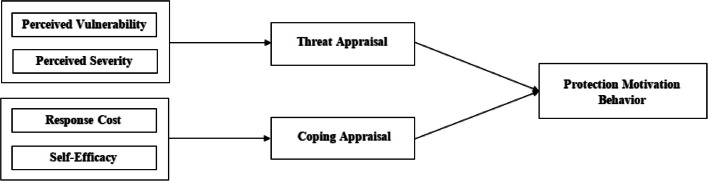
Protection motivation theory

While UTAUT2 provides a comprehensive framework for understanding users' behavioural intentions and technology usage, it has several limitations [2]. Cognitive perspectives are widely used to explain behavioural patterns and variables that may influence a protective or preventive action. These theories examine cognitive behaviour change and share the presumption that attitudes, beliefs, expectations of future events and outcomes are important factors for health-related behavior [72]. Protection Motivation Theory (PMT) is a social-cognitive model that identifies behavior [69]. PMT was presented by Rogers [84] as a theory for explaining the influence of fear appeals. It is commonly used in the health domain, but it has increasingly gained popularity in the information security domain [16, 99]. PMT examine the motivational reasons for adopting protective measures and breaks them into threat and coping appraisal. In the context of BYOD, the threat appraisal refers to the perceived vulnerability as a possibility of a security incident and the perceived severity as the impact of outcomes resulting from a security incident [27]. The coping appraisal refers to the individual's evaluation of how well he/she can manage in the given situation. The coping appraisal process consists of response costs and self-efficacy, as shown in Fig. 2.

**Fig. 3:**
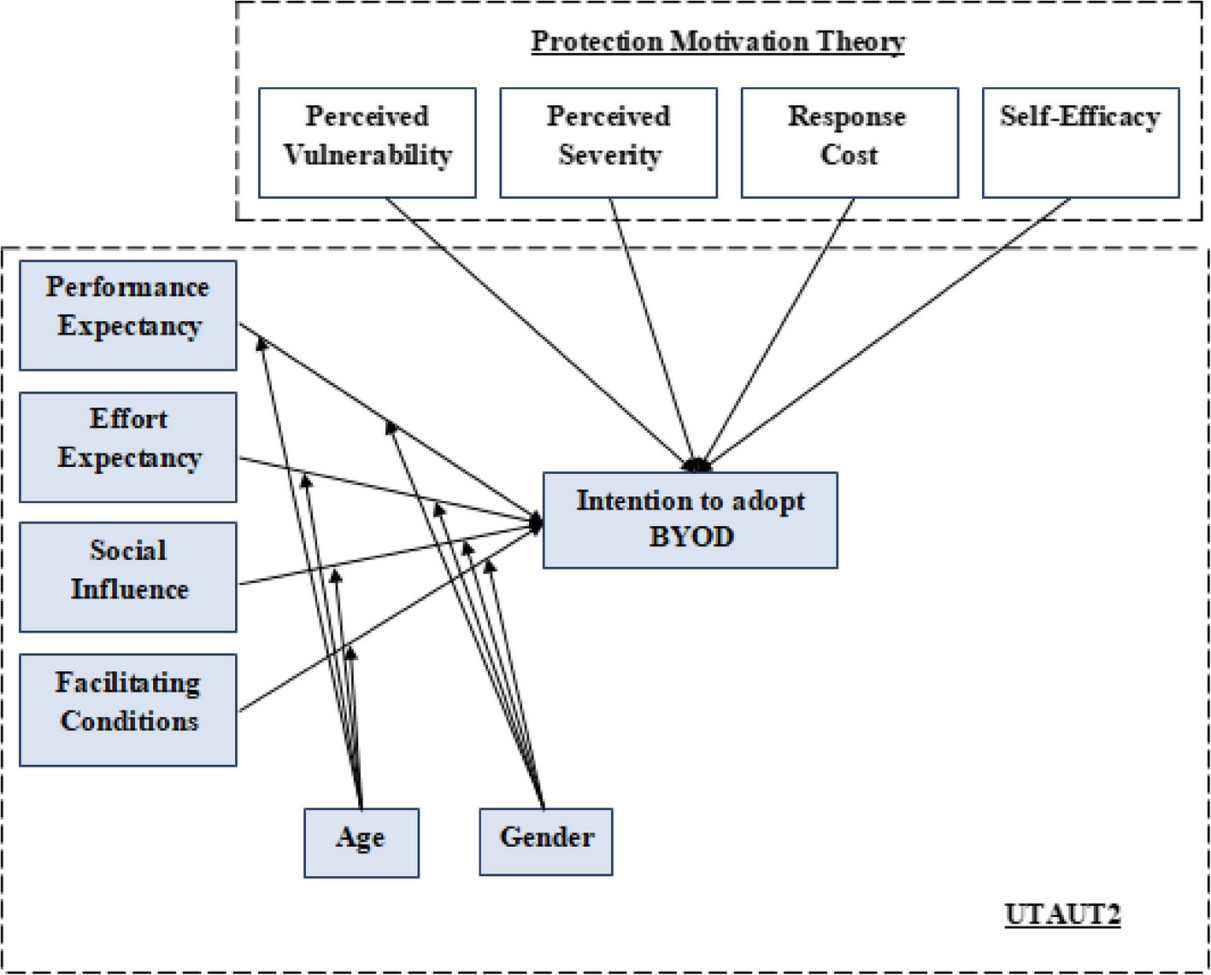
The theoretical model

This research's proposed hybrid model is presented in Fig. 3. The UTAUT model forms the backbone for the theoretical framework for this study. Additional factors were added to the base model to improve its performance regarding explaining the consumer's behaviour. Perceived vulnerability and perceived severity represented the threat appraisal and; response cost and self-efficacy represented coping appraisal. In this study, all additional hypotheses have a negative affect on behavioural intention except self-efficacy.

The present study makes slight modifications to the model. In this research, the focus is on behavioural intention. Since the dependent variable is behavioural intention, and the adoption is pre-implementation, there is no sense to measure the use of technology. Age and gender will moderate all relationships of UTAUT due to the novelty of BYOD and the pre-implementation research, experience and voluntariness of use as a moderator had dropped.

### Research objectives

The main research objective was to propose a hybrid model of intention to adopt BYOD amongst Pakistani doctors. Therefore, to accomplish the main objective, the following specific objectives were also formed:

- To identify the determinant factors for intention to adopt BYOD among Pakistani doctors
- To identify the moderating effect of age and gender on the intention to adopt BYOD
- To develop the hybrid model of behavioural intention to adopt BYOD among Pakistani doctors
- To validate the proposed model of intention to adopt BYOD through practitioners

### Hypothesis development

In this study, the hybrid model was proposed as the theoretical framework. The hypotheses are divided into two categories; main (direct) relationships and moderating relationships. In this study, Intention to Adopt BYOD (IAB) is the dependent variable. Behavioural intention is the strength of a user's intention to achieve a specific behaviour; it indicated a human's desire to adopt a technology [30, 101].

Performance Expectancy (PE) is a key predictor of behavioral intention in technology adoption across various fields, including healthcare, education, and commerce. It reflects the perceived benefits of technology in enhancing efficiency and outcomes. Research by [11] and [31] shows that PE significantly influences the adoption of Business Intelligence (BI) systems and blockchain in healthcare, emphasizing improved decision-making and operational efficiency. Studies by [90] further highlight how PE drives Bring Your Own Device (BYOD) and mobile device adoption by promoting mobility and convenience. In education, [50] underline PE’s role in providing resource access and bridging knowledge gaps. Studies have shown that PE significantly influenced behavioural intention to adopt technology in healthcare [3, 32, 81, 105]. In BYOD, PE is defined as how adopting the technology will influence users to perform activities [101, 102]. It shows the practical value for users adopting the technology, identified in different technology acceptance models, such as perceived usefulness in the TAM, extrinsic motivation in the Motivational Model (MM), and relative advantage in the Innovation Diffusion Theory (IDT).

Overall, these studies illustrate PE’s importance in technology adoption, particularly where clear benefits exist, such as faster decision-making and enhanced collaboration. In healthcare, PE encapsulates advantages like real-time patient data access and workflow integration, making it a crucial element of frameworks like the Unified Theory of Acceptance and Use of Technology (UTAUT). PE influences technology adoption by enhancing user acceptance, facilitating collaboration, and empowering staff through the use of preferred devices. Positive experiences with personal devices lead to faster decision-making and better integration into workflows, making users more likely to embrace BYOD policies. In this study, PE is a doctors' perception of the degree to which the doctors’ believe that using a BYOD will help him/her to attain gains in job performance. When doctors believe that using BYOD can empower them to improve healthcare capabilities, doctors' are likely to adopt the technology. Thus, PE was measured with five questions that focused on the intention to adopt BYOD. Therefore, the researcher hypothesized that:

- H1: Performance expectancy has a significant influence on the doctors' intentions to adopt BYOD in Pakistan

Effort Expectancy (EE) is a crucial predictor of behavioral intention in technology adoption, particularly in professional environments, acknowledged as the extent of ease linked to a consumers' technology utilization [5]. Research by [11] and [31] shows that healthcare professionals are more likely to adopt technologies like Business Intelligence (BI) systems and blockchain when they find them intuitive and easy to use. EE has a significant role in predicting behavioural intention in healthcare settings [32, 60, 86] significance of PEOU is a related factor to EE, as a predictor of behavioural intention, confirmed by other studies [7]. Another study [59] identified that healthcare professionals' behavioural intention for Electronic Health Record (EHR) system affected perceived ease of use rather than perceived usefulness. Furthermore, studies by [90] indicate that EE is vital in the adoption of BYOD policies and mobile technologies. Users prefer systems that integrate smoothly into their workflows without extensive training. It is also essential to recognize that the significance of EE can vary across different contexts and user demographics. A study by [50] found that, contrary to expectations, EE did not significantly affect medical students' decisions regarding Wi-Fi adoption. This outcome might be attributed to their prior familiarity with the technology and its functionalities, indicating that users' existing knowledge can influence their perceptions of new systems.

In the specific context of BYOD adoption within healthcare environments, the relevance of EE becomes even more pronounced. Healthcare professionals often operate under intense pressure, managing critical tasks that require efficiency and quick decision-making. Therefore, any perceived complexity associated with new technologies or a steep learning curve can significantly hinder their adoption. This reality highlights the necessity of developing BYOD systems that prioritize user-friendliness and can be easily incorporated into daily routines. Thus, it is essential to develop user-friendly BYOD systems that fit seamlessly into daily routines, promoting smoother adoption and enhancing efficiency in healthcare settings. The EE was measured with four questions that focused on the intention to adopt BYOD. Thus, the hypothesis of this research on the impact of the EE on behavioural intention among Pakistani doctors is:

- H2: Effort expectancy has a significant influence on the doctors' intentions to adopt BYOD in Pakistan

Social influence (SI) refers to how significant others' opinions can influence the individual's behaviour regarding new technology [101]. Recent studies emphasize that SI, shaped by peer influence, organizational culture, and societal norms, significantly impacts behavioral intention to adopt new technologies. SI is described as the extent to which a users' decision is affected by others' perceptions [93]. The construct of SI in the UTAUT model adopted from the TRA, TAM2, TPB/DTPB, and C-TAM-TPB; social factors (MPCU); and image (IDT) [62]. Previous research has shown [33] revealed that SI significantly influenced health professionals’ acceptance of mobile-based clinical guideline applications in resource-limited settings, highlighting the importance of colleagues and organizational culture in driving adoption. Similarly, studies by [11], and [31] explored the adoption of BI systems, blockchain, AI-driven healthcare services, and EHR systems, demonstrating that SI is a critical enabler for fostering acceptance, especially in environments requiring collaboration and teamwork. Additional research by [2] and [50] further reinforces that SI significantly drives adoption through peer recommendations and shared expectations. While studies like [90] suggest that SI may have limited impact in more autonomous professions, such as healthcare and military settings, they acknowledge that SI can complement other factors such as performance expectancy and facilitating conditions in promoting adoption. A study by [81] found that SI significantly influenced emergency information systems. However, several findings have concluded that because of context, respondents traits, and the technologies investigated, SI did not affect behavioural intention in the healthcare context [92]. The disagreement in the literature about the effect of SI on behavioural intention in healthcare helps this research investigate its significance among Pakistani doctors. These findings collectively justify the inclusion of SI as a predictor in BYOD adoption research, as it captures the influence of peer dynamics, leadership endorsement, and organizational culture in encouraging the acceptance and integration of personal devices in healthcare workflows.

The SI was measured with three questions that focused on the intention to adopt BYOD. The hypothesis is as follows:

- H3: Social influence has a significant influence on the doctors' intentions to adopt BYOD in Pakistan

Facilitating Conditions (FC) are consistently identified as a significant predictor of behavioral intention and technology adoption in UTAUT-based studies. FC refers to the availability of organizational and technical resources, including IT support, infrastructure, and policies, that enable users to adopt and effectively use a technology [101]. Studies such as [11] and [31] highlight the importance of FC in healthcare, demonstrating that robust IT systems and accessible resources significantly enhance the adoption of BI systems and blockchain technology. Similarly, [50] found that FC strongly influences both behavioral intention and actual use of Wi-Fi systems among medical students, indicating the critical role of a supportive environment in fostering technology use.

In resource-constrained settings, [75] showed that FC is essential for the adoption of EHR systems by healthcare professionals, emphasizing that technical support and infrastructure are pivotal in overcoming barriers. Studies like [110] also affirm that FC plays a vital role in BYOD adoption. Facilitating Conditions (FC) are crucial for predicting behavioral intention and technology adoption, particularly in healthcare. Research on the hospital's technical support and training substantially impacted HIS's adoption [18, 38]. Research indicates that strong FC enhances the adoption of technologies like business intelligence systems, blockchain, electronic health records (EHR), and Bring Your Own Device (BYOD) practices. Studies show that supportive environments significantly influence technology use among medical professionals and students, helping them overcome barriers and effectively integrate personal devices into their workflows. These findings collectively underscore that facilitating conditions are critical for the successful adoption of BYOD in healthcare, where robust IT support, clear policies, and training programs can help professionals overcome technical challenges and ensure efficient use of personal devices in their workflows. The FC measured with three questions that focused on the intention to adopt BYOD. Hypothesis for FC is:

- H4: Facilitating conditions has a significant influence on the doctors' intentions to adopt BYOD in Pakistan

The Perceived Vulnerability (PV) is the user's opinion about the probability that the threat will appear [65]. PV plays a significant role in influencing compliance behaviors within BYOD practices, largely through the process of threat appraisal. Research conducted by [97] explores BYOD security compliance through the lens of Protection Motivation Theory (PMT), identifying threat and coping appraisals as key predictors of compliance intentions. The study also highlights additional factors, such as mixed device usage and the visibility of company surveillance. Similarly, [4] investigate how perceived vulnerability, response efficacy, and subjective norms affect protective behaviors in BYOD environments. They suggest targeted interventions aimed at strengthening security practices among employees. Both studies emphasize that awareness of potential risks is a strong predictor of compliance intentions and protective behaviors, underscoring the necessity of enhancing risk awareness to promote secure practice. Additionally, [76] connect perceived vulnerability to security awareness and risk perception within their threat appraisal framework. Their study focuses on BYOD compliance in the public sector, establishing relationships between security behaviors and essential factors like policy clarity, self-efficacy, and psychological ownership, while emphasizing the critical role of training and support.

Individuals who believe their information system resources are vulnerable to an information system attack are more likely to take preventive measures [48]. Employees are vigilant about whether their data is secure, mainly using their own devices for work tasks. Hence, personal privacy and data confidentiality for using their mobile device is the most critical concerns [21]. In this context, it is possible to disclose employees' private details to an unapproved entity. If employees think his/her data security is not assured, it will stop them from accepting BYOD. The PV measured with three questions; the researcher proposed the following hypothesis:

- H5: Perceived vulnerability has a negative influence on the doctors' intentions to adopt BYOD in Pakistan

Perceived Severity (PS) refers to the degree of threat from unhealthy behaviours [84]. PS is observed as the degree to which a consumer thinks that risk impacts on his/her device would be dangerous [95]. However, there are different outcomes in the personal computing domain context [74]. If an employee uses his/her device instead of the company computer, the user's security risk can be a significant problem.

Perceived severity is a significant component of threat appraisal that influences compliance behaviors in Bring Your Own Device (BYOD) practices by highlighting the potential consequences of security breaches. Tu et al. [97] incorporated perceived severity into their Protection Motivation Theory (PMT) framework, demonstrating its influence on compliance intentions alongside other relevant factors. Similarly, [4] identified it as a crucial determinant of behavioral intentions in BYOD environments. In general, users considering adopting BYOD may act differently based on their PS [113], the PV measured with three questions. Consequently, the researcher proposed the following hypothesis.

- H6: Perceived severity has a negative influence on the doctors' intentions to adopt BYOD in Pakistan

Response Cost (RC) which refers to the perceived effort, inconvenience, individuals' costs when required to introduce a security policy safeguard measure, or time required to engage in protective behaviors, is vital for compliance with BYOD [115, 117]. It covers the costs of taking adaptive coping measures, like resources, time, commitment, inconvenience, complexity, comprehension, or other negative impacts on adopting the BYOD security plan [24]. If the expenses surpass the benefits, it is unlikely that the consumer will attempt any coping action [98]. RC applies to the costs affiliated with practising defensive behaviour. It has demonstrated a crucial part in personal computing, such that changes in perceived RC adversely affect intentions to implement security behaviours [95].

Moreover, [97] recognized response cost as a barrier to compliance intentions within the coping appraisal framework of Protection Motivation Theory (PMT). Al-Harthy and Ali [4] further supported this notion by showing that employees are less inclined to adopt protective behaviors when they perceive the effort as high, advocating for security measures that are user-friendly to foster compliance. When RC is high, individuals will be less likely to engage in a given protective behavior [96]. RC was found to negatively affect adopting anti-plagiarism software [65], BYOD practices [29] and online security behaviours [96]. Posey, Roberts & Lowry explained RC as perceived drawbacks such as expenses, disruptions, difficulties and likely adverse effects that users could incur [80].

Contrary to previous studies' findings, if a person agrees that the proposed preventive behaviour is appropriate, the cost of responding is justified [106]. A study by showed that the RC to mobile device users has no substantial impact on defending their device from data violations [44]. The study by [76] suggests that when organizational policies are clear and well-supported, the perceived effort or difficulty associated with compliance—conceptualized as response cost—is likely reduced. By streamlining processes and minimizing complexity, organizations can foster an environment where employees are more inclined to adhere to BYOD security measures, thereby enhancing overall compliance and security outcomes. If consumers of devices are assured in their self-efficacy against security attacks, RC would not significantly impact their defensive behaviour. Thus, the RC was measured with three questions; the researcher proposed that:

- H7: Response-cost has a negative influence on the doctors' intentions to adopt BYOD in Pakistan

Self-Efficacy (SE) is an individual's confidence in their capability to present behaviour [24]. It originated from the social cognitive theory, which alluded to an individual's belief in responding. However, according to social cognitive theory, people with stronger self-confidence in their skills can begin challenging behaviours. An individual's belief in his or her skills to complete a behaviour has positively influenced the mobile health system [117]. Alhelaly et al. [10] underscored the significance of self-efficacy as a vital factor in protection motivation, illustrating its role in mitigating privacy concerns and promoting compliance with BYOD policies. Similarly, [97] incorporated self-efficacy into their PMT framework, demonstrating that heightened self-efficacy enhances employees' coping abilities and strengthens their commitment to security measures. Al-Harthy and Ali [4] highlighted self-efficacy as a key determinant of behavioral intentions and protective behaviors in BYOD contexts, emphasizing the necessity of confidence-building strategies. These studies illustrate the critical importance of self-efficacy in BYOD security practices, urging organizations to prioritize the development of employee confidence through comprehensive training, robust support, and clear policies. Crossler, Long, Loraas, & Trinkle examined the factors determining whether employees follow BYOD policies through the PMT lens. They found that SE of BYOD is the salient factor for adopting BYOD policy [28].

Besides, the results obtained from several studies of PMT showed that SE might be the powerful predictor of intention to adopt preventative measures [112]. SE signifies an individual's ability to perform protection behavior [52]. By contrast, another study suggested the insignificant effects of SE [34]. Therefore, further empirical evidence is required for an improved understanding of this issue. In this research, SE refers to an individual's ability to implement security measures specified by a security policy to reduce the risk posed by BYOD adoption. Also, it can influence one's desire to accept BYOD, such as belief and desire to improve the adoption of mhealth, thus enhancing the overall attitude towards BYOD. Therefore, the SE was measured with four questions that focused on the intention to adopt BYOD. We propose the following hypothesis:

- H8: Self-efficacy has a significant influence on the doctors' intentions to adopt BYOD in Pakistan

Generally, different people have different perceptions about a particular aspect, or they understand and associate it uniquely based on their distinctive attributes, such as age, gender, preferences and experiences [47]. A moderator variable is a third variable affecting the relationship between the dependent and independent variables [85]. Within the study's contemporary context, two moderators were examined, age and gender.

Age moderated the relationship between performance expectancy, effort expectancy, social influence, facilitating conditions and behavioural intention. Elderly consumers tend to have more problems understanding new or complicated information, affecting their learning of new technologies [102]. In previous technology adopted research, age was assumed as a moderator. However, the influence depended on context, and results were not consistent across different settings. Research revealed that elderly individuals are less willing to adopt e-health technologies [32]. However, research in a healthcare setting, age did not significantly influence healthcare professionals' willingness to accept Health Information System (HIS). Research revealed that elderly individuals are less willing to adopt e-health technologies [32]. However, research in a healthcare setting, age did not significantly influence healthcare professionals' willingness to accept HIS. The effect of age on technology adoption has been well examined in a commercial context, but a lack of studies apply to healthcare settings [73]. The current study hypothesized the effect of age as the following:

- H9: Age will moderate the relationship between performance expectancy, effort expectancy, social influence and facilitating conditions on the intention to adopt BYOD

**Fig. 4:**
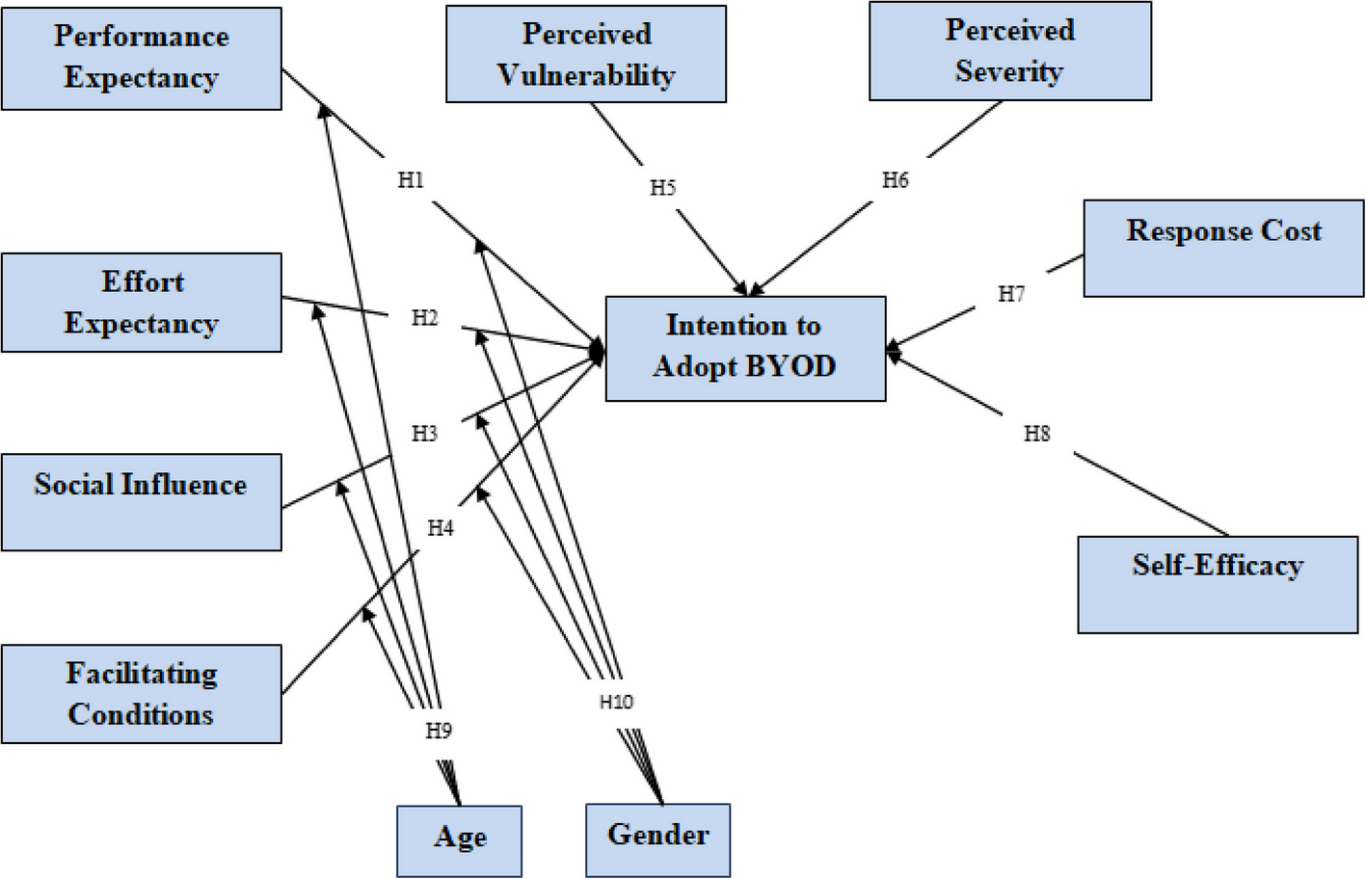
The conceptual hybrid model of the study

Gender is vital while examining performance expectancy, effort expectancy, social influence, facilitating conditions on BI [102]. Studies examining the role of gender indicate how personality characteristics and gender variation are essential to understanding and using emerging technologies. The role of gender cannot be ignored in the evaluation of the acceptance of technology. The original UTAUT model investigated gender as a moderator and found its significant impact on the relationship between facilitating conditions to intention to adopt [101]. Another study also found that females are less interested and likely to adopt e-health technology [32]. To evaluate the impact of gender as a moderator on the relationship between facilitating conditions and intention to adopt the hypothesis is:

- H10: Gender will moderate the relationship between performance expectancy, effort expectancy, social influence and facilitating conditions on the intention to adopt BYOD. The 08 main research hypotheses and 02 moderator hypotheses are summarized in Table 1 in a tabular format; the hybrid research model is also presented in Fig. 4.

**Table 1:** Research hypotheses

Main Hypotheses		
Code	Construct	Hypotheses
H1	Performance Expectancy	Performance expectancy has a significant influence on the doctors' intentions to adopt BYOD in Pakistan.
H2	Effort Expectancy	Effort expectancy has a significant influence on the doctors' intentions to adopt BYOD in Pakistan
H3	Social Influence	Social influence has a significant influence on the doctors' intentions to adopt BYOD in Pakistan.
H4	Facilitating Conditions	Facilitating conditions has a significant influence on the doctors' intentions to adopt BYOD in Pakistan.
H5	Perceived Vulnerability	Perceived vulnerability has a negative influence on the doctors' intentions to adopt BYOD in Pakistan.
H6	Perceived Severity	Perceived severity has a negative influence on the doctors' intentions to adopt BYOD in Pakistan.
H7	Response-Cost	Response-cost has a negative influence on the doctors' intentions to adopt BYOD in Pakistan.
H8	Self-Efficacy	Self-efficacy has a significant influence on the doctors' intentions to adopt BYOD in Pakistan.

Moderators		
Code	Constructs	Hypotheses
H9	Age	Age will moderate the relationship between PE, EE, SI and FC on the intention to adopt BYOD.
H10	Gender	Gender will moderate the relationship between PE, EE, SI and FC on the intention to adopt BYOD.

## Methods

### Research design

The quantitative approach was applied to investigate the relationship between exogenous and endogenous variables based on the research's nature and philosophy. Furthermore, the cross-sectional design was adopted due to time and financial constraints. Notably, the cross-sectional approach was also found appropriate due to the academic nature of the present study. Henceforth, the primary data was collected using a survey method for statistical interpretations, inferences, and conclusions. The survey method's significant benefit includes time flexibility for respondents, allowing them to fill questionnaires at their convenience. This study closely adhered to the STROBE statement for cross-sectional studies for the reporting of this study [100, 107].

### Sample size

The sample size for this study was determined based on Sekaran guidelines [88], which recommend a sample size of 384 for populations exceeding 75,000 to achieve a 95% confidence level and a ± 5% margin of error. With a total population of 183,800 registered doctors in September 2019, this study adopts the recommended sample size of 384, as outlined in Table 2 below.

**Table 2:** Survey sample size

Population Size (N)	Sample Size (S)
10,000	370
15,000	375
20,000	377
30,000	379
40,000	380
50,000	381
75,000	383
1000,000	384

Source: [88]

The sample size needed to be ten (10) times the study's model's independent variables for the analysis, which deals with the variables' interrelationship [46]. So, the obtained questionnaires were adequate to carry out further research throughout the study. Since the maximum count of variables used in this study was 8, this study's sample size needed more than 80 respondents. The 249 functional questionnaires fulfil the threshold for analysing the relationship between the variables of this study's conceptual hybrid model. Considering several studies in the Information System(IS) field, out of 550 questionnaires distributed, 249 (45.2 per cent) available responses seemed adequate for further study [66, 77, 104].

### Facebook as a sample frame: virtual snowball sampling

**Fig. 5:**
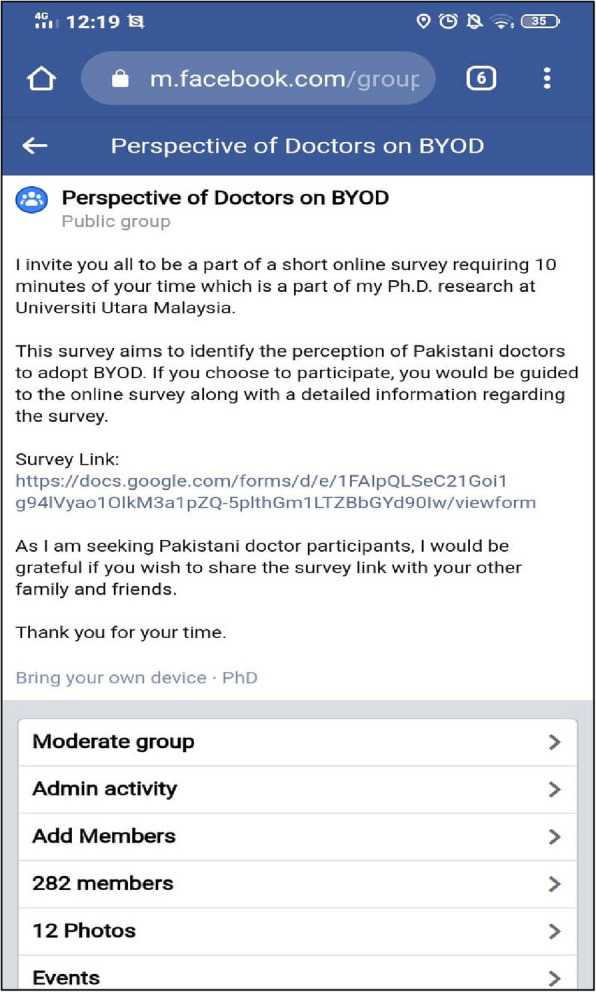
Sample of Facebook group "perspective of doctors on BYOD"

This study applied to Pakistani doctors from different cities, governments, and private hospitals registered under the Pakistan Medical and Dental Council (PMDC). The respondents were asked to provide their medical license number to verify respondents as licensed doctors of Pakistan. To ensure privacy and anonymity, the license numbers were immediately anonymized by replacing them with unique, randomly generated codes. No personally identifiable information was stored or used in the analysis, and all data processing adhered to strict confidentiality protocols. These measures ensured that the respondents' identities remained protected throughout the research. The license number of doctors provided in the survey form was verified on the website of PMDC. The sample of a doctor's verification through the PMDC website is provided in Fig. 5. The total number of registered doctors under the PMDC in Pakistan is reported as one hundred eighty-seven thousand nine hundred and seventy (187,970) [79].

Doctors in Pakistan do not have an official e-mail ID and database. The PMDC provide only the total number of registered doctors and their license details. It is not feasible to identify them with a convenient sampling method. Several researchers have used online surveys to access the "hard to reach" population [57, 111]. One study contrasted a distinct recruited community with open recruitment to study young smokers. Another study set up a link to obtain confidential recreational drug users. Finally, Social Networking Sites (SNSs) used in social studies can ease the research to a dual extent. Primarily it is a convenient medium to classify the most difficult to reach respondents and increase the sample size. Following this, it can reduce the number of obstacles connected with online data collection techniques.

The first question the researcher had to deal with was getting in touch with the doctors in Pakistan. To the researcher's understanding of this, no sampling frame of doctors is available in Pakistan. More than 161 million people are using phones in the country, and penetration density is 76.76%. PTA [82], almost everybody is on Facebook. Many doctors living in Pakistan would also do so. So, why not try to find them in that virtual space?

The doctor's reluctant behaviours are the second concern. First, participants sometimes faced the risk of participating with very little information about the study's aim. Facebook also allows contact person-to-person through their profiles. There is no intermediation to allow the researcher to reach participants directly. We understand that people primarily use Facebook in their free time rather than regular work hours. So, approaching participants via Facebook may decrease resistant behaviours due to work or job duties. Considering this benefit, the researcher decided to continue the research using Facebook as the leading platform to recognize participants and gather data.

The first phase involved the development of an ad hoc Facebook profile for study purposes. The profile included information on the research; this provides access to the social network to start the research process. However, a profile is a connection between the researcher and the participants. The profile serves to build trust and respect for the individual to participate without scepticism. Accordingly, a profile should include all the details that the researcher considers appropriate for participants.

The second step consisted of the subject research itself. In this case, the researcher needed to find Pakistani doctors; one way to approach the doctors was to identify the Facebook groups associated with the doctor's community. It identified that doctors have Facebook groups related to further studies such as Fellow of College of Physicians and Surgeons (FCPS), Intermediate Module (IMM) and the United States Medical Licensing Examination (USMLE). The researcher identified different groups; specifically,"FCPS Prep. Batch (2005–2010)"had 171,698 members; most of them are Pakistani doctors. With the permission of a Facebook group admin, the researcher posted a message on the group's wall for every member to see, including the survey link. The researcher also created their own Facebook group named"Perspective of Doctors on BYOD"and successfully added more than 280 doctors from all over Pakistan. The sample of the group is presented in Fig. 5.

**Fig. 6:**
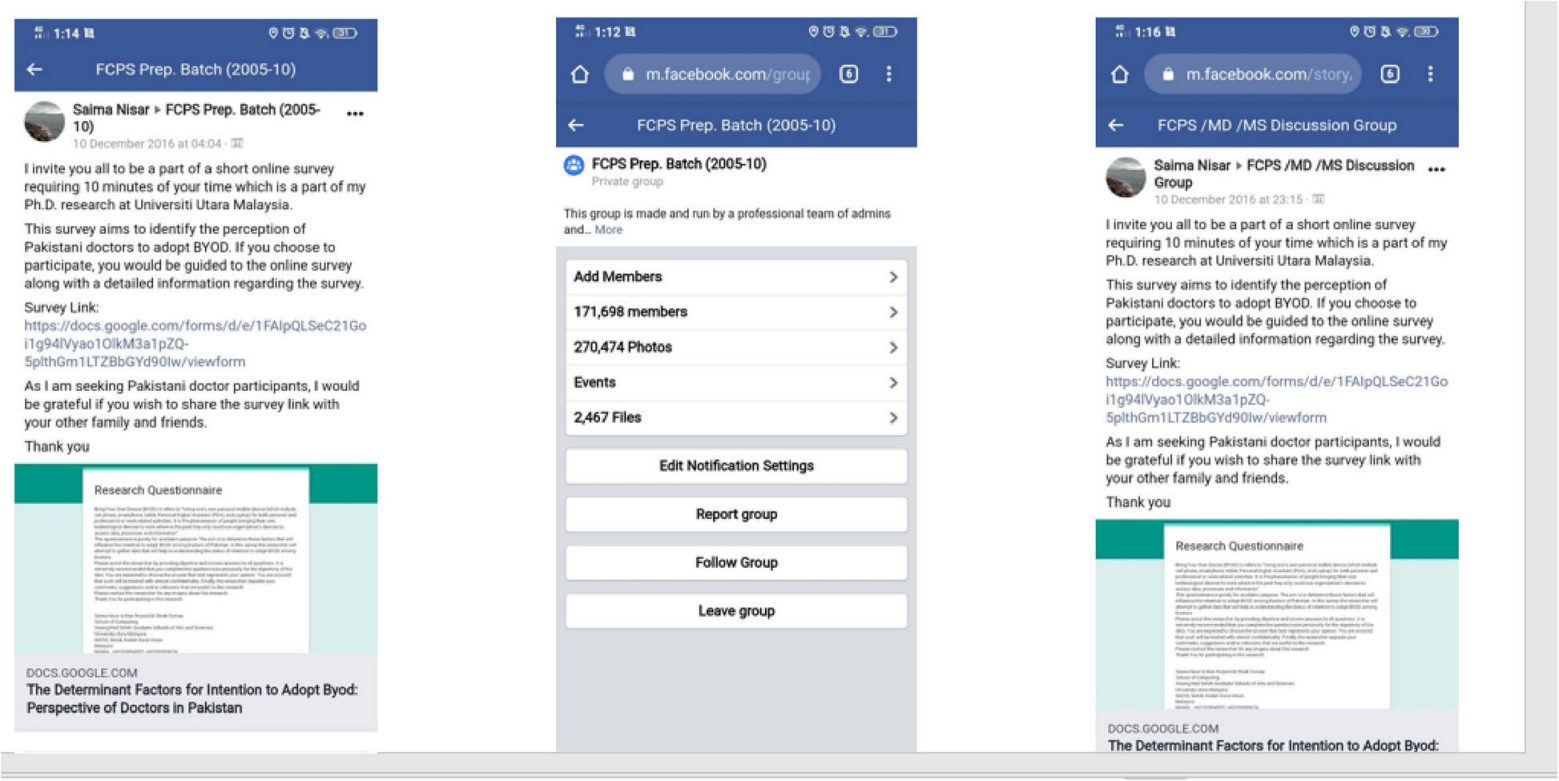
Sample of Facebook group for recruiting the participants

One way to approach the participants was to contact all groups' members, send them private messages, and ask if they are registered medical doctors from Pakistan and interested in participating in the survey. The researcher also posted a message on their own group's wall for every member to see. The example of Facebook groups' and Facebook group members' profiles is provided in Fig. 6. Furthermore, the researcher decided to extend the sample size, asking every respondent if they have a connection with an individual who could fit the sample criteria and participate in the study.

Regarding the accuracy of the survey, a significant concern associated with employing virtual sampling is the reliability of the data achieves through the internet [41]. In this research, the researcher regarded the use of a different technique to verify the information. Initially, the researcher asked doctors about the demographic information; secondly, the researcher asked the doctors' to provide their medical license number to verify the data's authenticity. The population reacts reliably within the virtual environment [36, 118].

**Fig. 7:**
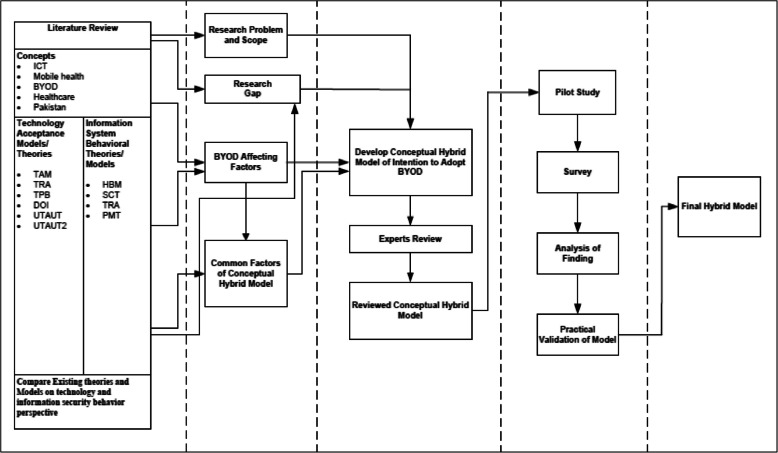
Phases in the research methodology

The processes involved in this study interacted with each other during the period of the study. This connection is outlined illustratively in Fig. 7. The primary focus of this study was the identification of the factors for the intention to adopt BYOD. Thus, to accomplish the objectives, sequences of steps were involved.

#### Phase I

A literature review was conducted in understanding the concept related to this study. The issues to be researched were defined through elicitation from the literature. Several facts from current articles and actual data established the research problems. The established key issues resulting from the literature study guided the research gaps, objectives, and scopes, as discussed previously in the first chapter.

#### Phase II

After identifying the research problem, gap, and scope of the study, the researcher moved on to the second phase. The study aimed to propose a conceptual hybrid model for the adoption of BYOD amongst Pakistani doctors. The factors identified and developed the proposed hybrid model through a comprehensive review of the existing theories and models.

#### Phase III

Phase III was used to confirm the determining factors. The identified factors in the previous phase, together with the components, were integrated to form the hybrid model. After proposing a conceptual hybrid model, the expert's review approach was utilized to identify factors. The expert review involved academicians of international universities. Experts looked into the proposed factors, terms used, and the logic of all components' connections and flow. They also ensured that the proposed hybrid model and survey instrument is readable. A piece of the instrument provided them to review the proposed hybrid model and annotate helpful comments to improve its design. In particular, e-mail services are utilized as the communication medium. Having been justified by the expert's comments, the researcher reviewed proposed behavioural intention factors to adopt BYOD amongst Pakistani doctors.

#### Phase IV

To ensure the proposed hybrid model is valid, phase IV performed in conjunction with a pilot study, survey, data analysis, and practical validation of doctors' model as field experts.

#### Phase V

In the final phase, all the previous phases' findings were concluded by revisiting and answering the research questions and objectives. Finally, this study comes out with a validated hybrid model through field experts (Doctors).

### Data collection

The study adapted validated scales from prior research to design a survey measuring eight variables: performance expectancy, effort expectancy, social influence, facilitating conditions, perceived vulnerability, perceived severity, response cost, and self-efficacy, along with two moderators: age and gender. Items were slightly reworded to align with the context of Pakistani doctors [54, 93, 101], some changes in wording made on these items. The questionnaire had two parts: demographic data collection and 31 items reflecting study factors, assessed on a 7-point Likert scale (1 = strongly disagree, 7 = strongly agree).

The survey was distributed via Google Forms to 550 doctors using private messages and Facebook to enhance the response rate. Follow-up reminders, as recommended in prior studies, were employed. Of the 550 distributed surveys, 278 responses were received (50.5% response rate). After excluding 29 responses lacking license numbers, 249 valid responses (45.2%) were used for analysis as indicated in Table 3 [68, 77]. This met the threshold for survey-based studies, where a response rate of 30% and a sample size of at least 10 times the independent variables are acceptable [45, 87].

**Table 3:** Response rate of the questionnaires

Response	Frequency
No. of distributed questionnaires	550
Returned questionnaires	278
Returned and usable questionnaires	249
Returned and excluded questionnaires	29
Questionnaires not returned	272
Response rate	50%
Valid response rate	45%

The target respondents were PMDC-registered doctors, verified through a buffer question. The study ensured the data's adequacy for analysis, as 249 valid responses exceeded the minimum requirement based on the study's eight variables. Despite doctors' busy schedules, the response rate was sufficient and comparable to similar studies in healthcare contexts.

SPSS was used to check for missing values, and no missing data were found. The dataset was complete, enabling thorough analysis of the hybrid conceptual model's variables.

### Statistical analysis

The preliminary data were coded, and the returned and functional questionnaires were entered via the SPSS in.csv format, and the initial analysis was carried out in the International Business Management (IBM) Statistical Package for Social Sciences (SPSS) version 25. Data screening included missing value analysis, outlier evaluation, normality, and multicollinearity test [94]. Missing values did not exist in the given dataset. Mahalanobis Distance (D2) was carried out in Multivariate outliers; 4 cases were identified as outliers and removed. With the exclusion of these cases, the maximum number of cases available for the analyses was 245. After initial screening, the data normality was checked using skewness and kurtosis. The outcomes of both Kurtosis and Skewness showing the data was not normally distributed. Notably, rigorous data quality control was implemented, and missing values was determined through comprehensive analysis using SPSS 25.

## Results

The participants' demographic information included age, gender, experience, medical speciality, hospital and working hours, mobile devices, type, brand, mobile at work, and time-related work is summarized in Table 4. Notably, rigorous data quality control was implemented, and it was determined that there were no missing values in the dataset, as confirmed through comprehensive analysis using SPSS 25.

**Table 4:** Descriptive analysis of demographic data

Demographic Variable	Category	(*N* = 249)	Percentage
		Frequency	%
Gender	Male	211	84.7
	Female	38	15.3
Age	Under 25 Years	20	8.0
	25—34 Years	180	72.3
	35–44 Years	24	9.6
	45—54 Years	9	3.6
	Above 54 Years	16	6.4
Medical Specialty	Family Physician	26	10.4
	General Internal Medicine	77	30.9
	Pediatric	21	8.4
	Surgical Specialty	32	12.9
	Ob/Gyn	12	4.8
	General Surgery	18	7.2
	Other	63	25.3
Experience	Less than 1 Years	20	8.0
	1–2 Years	82	32.9
	3–5 Years	74	29.7
	6–10 Years	29	11.6
	11–15 Years	16	6.4
	16–20 Years	8	3.2
	Above 20 Years	20	8.0
Hospital	Public	156	42.7
	Private	167	45.8
	Both	40	11.0
Working Hours	0–10 h	14	5.6
	11–20 h	19	7.6
	21–30 h	10	4.0
	31–40 h	29	11.6
	41–50 h	58	23.3
	51 + hours	119	47.8
Mobile Device	Yes	249	100
	No	0	
Device Type	Cellular Phone	14	5.6
	Smartphone	224	90.0
	Tablet	11	4.4
Device Brand	Apple	50	20.1
	Samsung	122	49.0
	Huawei	38	15.3
	Q-Mobile	7	2.8
	Lenovo	4	1.6
	HTC Mobile	2	0.8
	Oppo	6	2.4
	Microsoft	2	0.8
	Sony	6	2.4
	Other	12	4.8
Mobile at Work	Yes	244	98.0
	NO	5	2.0
Time on Mobile	None to 30 Minutes	9	3.6
	1–2 h	72	28.9
	3–4 h	111	44.6
	5–6 h	37	14.9
	7 h or more	20	8.0
Time Work-Related	None to 30 Minutes	79	31.7
	1–2 h	135	54.2
	3–4 h	23	9.2
	5–6 h	8	3.2
	7 h or more	4	1.6

### Measurement model

The Partial Least Squares Structural Equation Modelling (PLS-SEM) has been suggested for data analysis in the case of non-normal data. This research applied the SmartPLS 3.0 software to examine inferential statistics [46, 83]. In the measurement model's assessment, also recognized as the outer model, all constructs are reflective. The measurement model analysis was performed based on construct reliability, converging, and discriminant validity. The measurement model measured the reliability using the Cronbach's alpha, and Composite Reliability (CR), values of 0.70 or higher for all constructs were considered acceptable [61]. The outer loading and the Average Variance Extracted (AVE) were considered to establish convergent validity.

Consequently, the approved cut-off value for outer loading is 0.70, while for AVE, it is 0.50 [47]. CR for all the constructs was above the threshold of 0.7 [46], and AVE for all the constructs was above 0.5. Table 5 summarizes the analysis of construct reliability and convergent validity of the current study.

**Table 5:** Analysis of measurement model

Construct	Indicator	Outer Loading	Cronbach’s Alpha	Composite Reliability	AVE	Convergent Validity
PE	PE1	0.922	0.963	0.971	0.871	Achieved
	PE2	0.939				
	PE3	0.936				
	PE4	0.931				
	PE5	0.938				
EE	EE1	0,947	0.961	0.972	0.896	Achieved
	EE2	0.956				
	EE3	0.963				
	EE4	0.919				
SI	SI1	0.896	0.827	0.897	0.746	Achieved
	SI2	0.938				
	SI3	0.744				
FC	FC1	0.885	0.899	0.937	0.832	Achieved
	FC2	0.942				
	FC3	0.910				
IAB	IAB1	0.970	0.968	0.979	0.940	Achieved
	IAB2	0.978				
	IAB3	0.962				
PV	PV1	0.893	0.906	0.941	0.842	Achieved
	PV2	0.927				
	PV3	0.933				
PS	PS1	0.905	0.914	0.946	0.854	Achieved
	PS2	0.943				
	PS3	0.925				
RC	RC1	0.897	0.857	0.912	0.776	Achieved
	RC2	0.861				
	RC3	0.884				
SE	SE1	0.942	0.968	0.977	0.912	Achieved
	SE2	0.967				
	SE3	0.970				
	SE4	0.941				

*PE* Performance Expectancy, *EE* Effort Expectancy, *SI* Social Influence, *FC* Facilitating Conditions, *IAB* Intention to adopt Byod, *PV* Perceived Vulnerability, *PS* Perceived Severity, *RC* Response Cost, *SE* Self-Efficacy

After convergent validity analysis, a test on discriminant validity was performed, the discriminant validity results based on the Fornell-Larcker criterion are shown in Table 6.

**Table 6:** Discriminant validity (Fornell-Larcker method)

	EE	FC	IAB	PE	PS	PV	RC	SE	SI
EE	0.946								
FC	0.838	0.912							
IAB	0.884	0.837	0.970						
PE	0.904	0.808	0.841	0.933					
PS	0.567	0.540	0.589	0.492	0.924				
PV	0.544	0.562	0.516	0.489	0.765	0.918			
RC	0.467	0.504	0.482	0.432	0.697	0.688	0.881		
SE	0.843	0.783	0.886	0.776	0.629	0.552	0.536	0.955	
SI	0.708	0.695	0.628	0.700	0.358	0.404	0.311	0.594	0.863

*PE* Performance Expectancy, *EE* Effort Expectancy, *SI* Social Influence, *FC* Facilitating Conditions, *IAB* Intention to Adopt BYOD, *PV* Perceived Vulnerability, *PS* Perceived Severity, *RC* Response Cost, *SE* Self-Efficacy

### Structural model

**Fig. 8:**
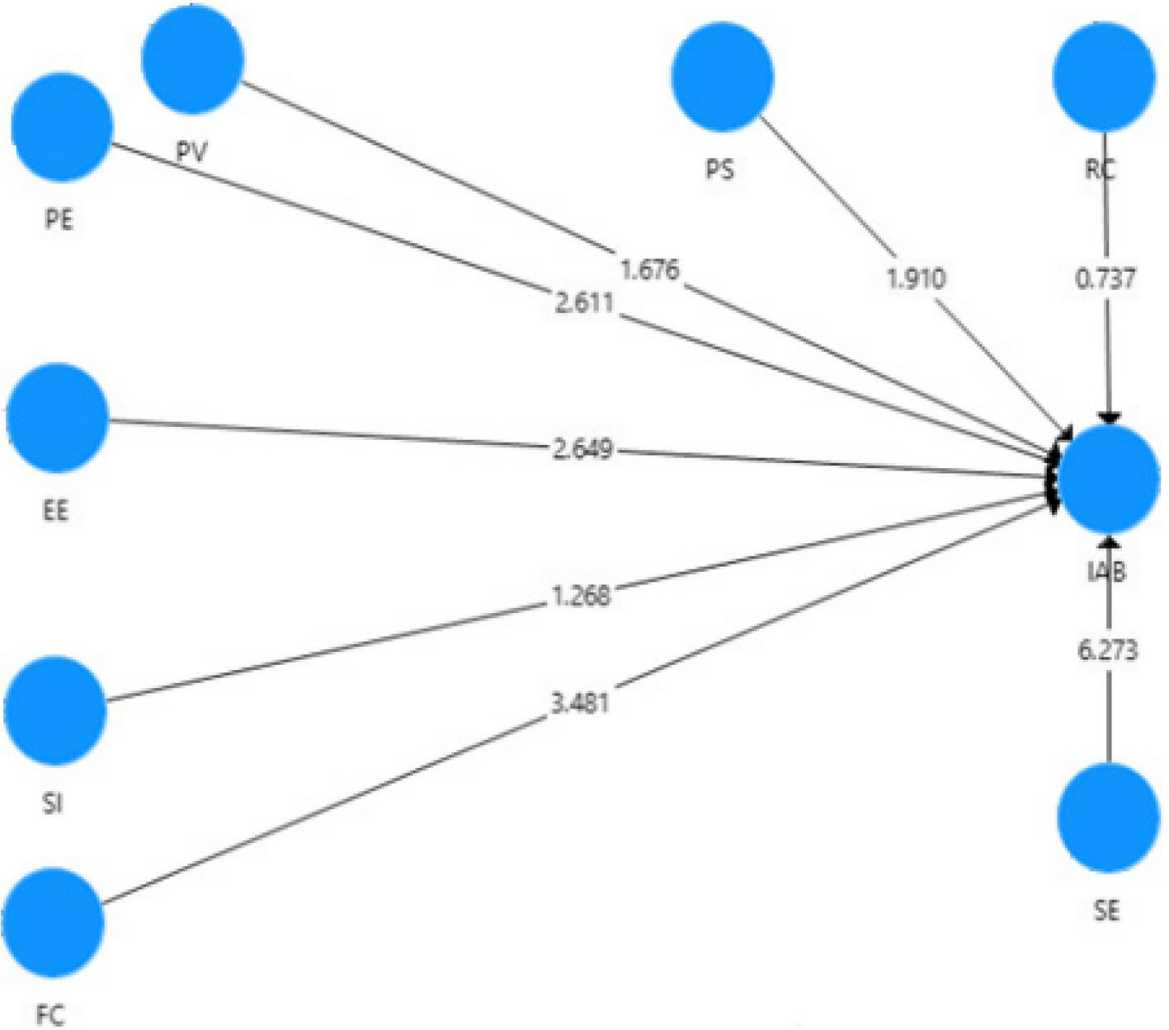
Structural model of direct relationships

The next step was to examine all the hypotheses, including primary and moderating relationships, the analysis of the structural model performed to examine the created hypotheses of the study. The analysis used a PLS bootstrapping algorithm of 5,000 samples for 245 cases to assess the importance of the path coefficient (ß) [46]. The hypothesized relationships were illustrated in Fig. 8. The Coefficient of Determination (R2) value represented a model's predictive accuracy and was measured as the squared correlation between a particular dependent variable's actual and predicted values [46]. The structural model used in this study explained 87% of the variance in behavioral intention to adopt BYOD. The Prediction Relevance (Q^2^) or Stone-Geisser's Q^2^ test reflects the model's ability to predict the measurement items of any dependent variable in model [43, 49]. The Q^2^ values for this study model were equal to 0.809, which was higher than the threshold limit, and supports that the path model's predictive relevance was adequate for the dependent variable.

Performance expectancy (ß = 0.158, *t*-value = 2.611, *p*-value = 0.005), effort expectancy (ß = 0.240, *t*-value = 2.649, *p*-value = 0.004), facilitating conditions (ß = 0.225, *t*-value = 3.481, *p*-value = 0.000), perceived severity (ß = 0.085, *t*-value = 1.910, *p*-value = 0.028) and self-efficacy (ß = 0.414, *t*-value *t* = 6.273, *p*-value = 0.000) had a positive significant influence on the doctors' intention to adopt BYOD. Additionally, perceived vulnerability (ß = −0.075, *t*-value *t* = 1.680, *p*-value = 0.047) had a slightly negative direct effect on intention to adopt BYOD. Table 7 shows H3 and H7 of the eight research hypotheses were rejected. The standardized path coefficients of all other relationships were significant at *P* < 0.05. Structural model statistics with effect size (f^2^) also provided in Table 7. The appropriate interpretation of the f^2^ effect size is 0.02 for small, 0.15 for medium, and 0.35 for a large effect [46].

**Table 7:** Hypotheses testing

Hypothesis	Relationship	ß	*T* Values		*P* Values*	Decision	f^2^
H1	PE—> IAB	0.158	2.611	0.005		Supported	0.034
H2	EE—> IAB	0.240	2.649	0.004		Supported	0.053
H3	SI—> IAB	−0.047	1.268	0.102		Not Supported	0.008
H4	FC—> IAB	0.225	3.481	0.000		Supported	0.094
H5	PV—> IAB	−0.075	1.680	0.047		Supported	0.015
H6	PS—> IAB	0.085	1.910	0.028		Supported	0.018
H7	RC—> IAB	−0.026	0.737	0.230		Not Supported	0.003
H8	SE—> IAB	0.414	6.273	0.000		Supported	0.310

significant at *p* < 0.05

### Moderators

The moderating analysis between the independent variables: performance expectancy, effort expectancy, social influence and facilitating conditions to the dependent variable intention to adopt BYOD were performed using PLS Multi-Group Analysis (PLS-MGA). The p-value for MGA should be below 0.05 to be considered significant.

The age was grouped into two categories: young adulthood 197 doctors with a percentage of 80.40% and middle adulthood 48 doctors with a percentage of 19.50%. Morris and Venkatesh suggested that individuals in their twenties (20's) and thirties are considered young, and those in their forties ('40 s) and above are considered old [71]. After conducting the MGA to examine the effect of age as a moderator, the relationship between performance expectancy, effort expectancy, social influence and facilitating conditions on the intention to adopt BYOD, from Table 8, it is clear that the differences between ß values of young adult and middle adult doctors are minor. Furthermore, the result of PLS-MGA also reveals that all p-values of differences between young adult and middle adult doctors are not significant, which calls for the rejection of H10, and as a result, age did not moderate those relationships.

**Table 8:** Multi-group analysis of age

Hypothesis	Relationship	ß Young Adult	ß Middle Adult	ß—diff (Young Adult- Middle Adult)	*t*-Value (Young Adult vs Middle Adult)	*p*-Value (Young Adult vs Middle Adult)	Decision
H9	EE—> IAB	0.504	0.582	−0.078	0.328	0.372	Not Significant
	FC—> IAB	0.316	0.354	−0.038	0.177	0.430	Not Significant
	PE—> IAB	0.213	−0.001	0.214	1.178	0.120	Not Significant
	SI—> IAB	−0.102	−0.027	−0.075	0.587	0.279	Not Significant

significant at *p* < 0.05

The MGA was carried out to investigate whether there is a significant difference between the two groups regarding the intention to adopt BYOD. The role of gender as the categorical moderator was examined; the study sample included 208 male doctors with 84.89% and 37 female doctors with a percentage of 15.10%. The relationships between performance expectancy, effort expectancy, social influence, and facilitating conditions to the intention to adopt BYOD were consistent between male and female respondents. The results do not show a significant moderating effect for gender, as presented in Table 9.

**Table 9:** Multi-group analysis of gender

Hypothesis	Relationship	ß Male	ß Female	ß—diff (Male—Female)	*t*-Value (Male vs Female)	*p*-Value (Male vs Female)	Decision
H10	EE—> IAB	0.503	0.607	−0.104	0.408	0.342	Not Significant
	FC—> IAB	0.321	0.31	0.011	0.051	0.480	Not Significant
	PE—> IAB	0.189	0.022	0.167	0.842	0.200	Not Significant
	SI—> IAB	−0.089	0.011	−0.100	0.654	0.257	Not Significant

significant at *p* < 0.05

### Practical validation

**Fig. 9:**
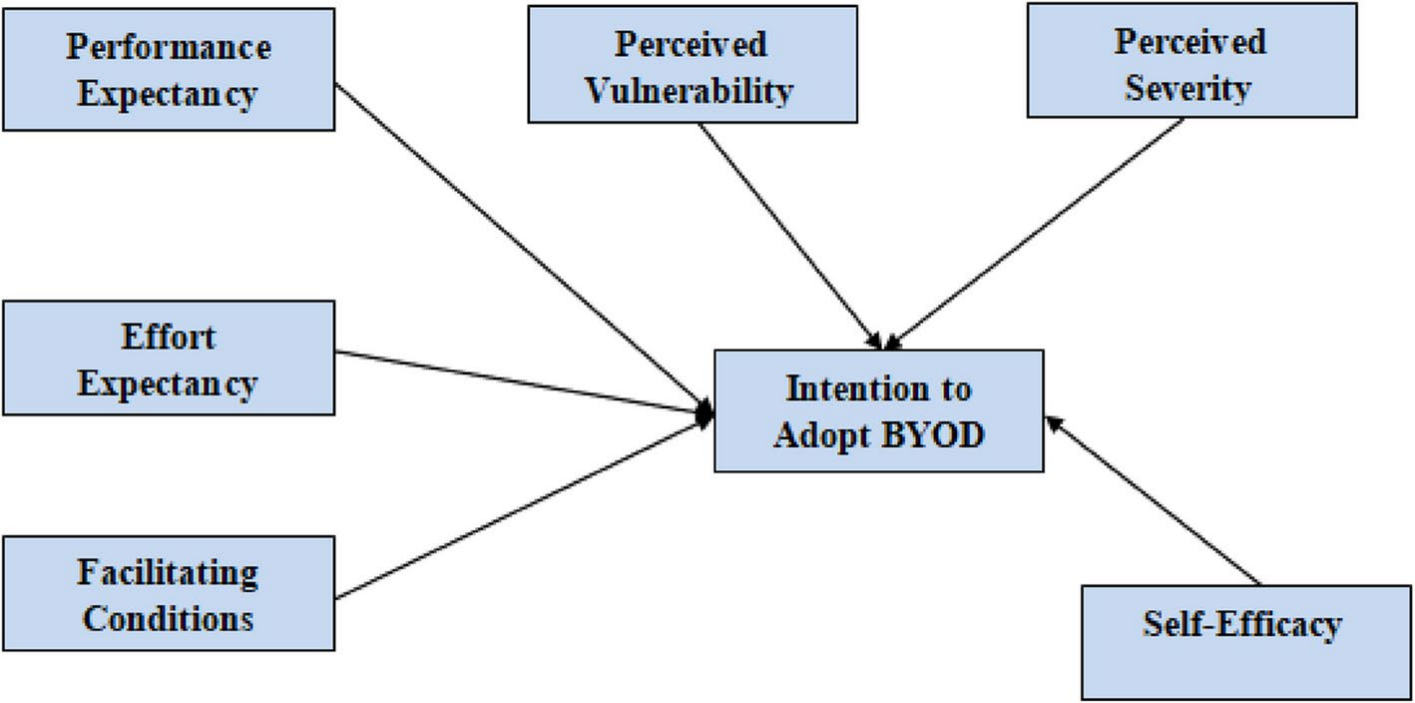
The hybrid model for doctors' intention to adopt BYOD

This study has produced recommendations based on the results. Considering the recommendations made by [40], the number of experts for validation should have at least two reviewers who are experts in the area be measured. The validation was performed by two expert doctors who have more than five years of experience. Both practitioners were agreed on the practicality of the recommendations and the hybrid model. Therefore, based on the preceding discussion, it is concluded that the hybrid model, as presented in Fig. 9, is valid and practical.

## Discussion

This research attempted to identify factors in adopting the BYOD among doctors in Pakistan, using a hybrid model. The structural model used in this study explained 87% of the variance in behavioral intention to adopt BYOD. Performance expectancy, effort expectancy, facilitating conditions and self-efficacy were positive predictors of behavioral intention, while perceived vulnerability and perceived severity affected the intention negatively. In this study, social influence and response cost were not associated with behavioural intention. The individual characteristics of age and gender did not significantly influence intention to adopt BYOD.

Performance Expectancy (PE) (*p* = 0.005) and Effort Expectancy (EE) (*p* = 0.004) emerged as significant predictors of the intention to adopt BYOD among doctors. These constructs, foundational to the UTAUT2 framework, collectively underscore the dual importance of perceived benefits and ease of use in technology acceptance [35]. PE demonstrated the fourth-strongest relationship with Intention to Adopt BYOD (IAB), highlighting its pivotal role in shaping adoption decisions [11]. PE underscoring that doctors operating in high-pressure environments place significant importance on the effectiveness and efficiency that BYOD can bring to their practice. BYOD enables doctors to efficiently manage medical records, track health information, access clinical histories, make informed decisions, and provide high-quality health services. The ability to access information 24 h a day, make quicker decisions, and increase service efficiency are compelling benefits that align with doctors' professional responsibilities [53]. These functionalities not only streamline workflows but also enhance patient care, which is paramount in medical practice.

Effort Expectancy (EE), on the other hand, pertains to the perceived ease of using BYOD technologies. The study found that EE is the third strongest predictor of the intention to adopt BYOD, highlighting its significant role alongside PE [2]. Doctors are more likely to embrace BYOD if they find the devices and applications user-friendly and easy to integrate into their daily workflows. Given the demanding and time-sensitive nature of medical professions, technologies that do not require extensive training or adaptation are highly valued. The significant positive relationship between EE and adoption intention suggests that minimizing the effort required to use BYOD can substantially enhance its acceptance among healthcare professionals.

The interplay between PE and EE suggests that doctors are motivated to adopt BYOD not only because they recognize its potential to enhance their professional capabilities but also because they believe it can be seamlessly integrated into their existing workflows without significant disruption. The significant influence of Performance Expectancy and Effort Expectancy on doctors' intention to adopt BYOD highlights the importance of designing and implementing technologies that offer clear performance benefits while ensuring ease of use. Healthcare organizations aiming to promote BYOD adoption should focus on enhancing the perceived usefulness of these devices and ensuring that they are user-friendly and seamlessly integrate into existing clinical workflows. By addressing both the functional and usability aspects, organizations can effectively foster a conducive environment for BYOD adoption among medical professionals.

Self-efficacy was found to have the most significant path coefficient and effect size, indicating the most substantial positive factor of intention to adopt BYOD. The current study indicates that doctors feel comfortable using their own devices and operate them with minimal assistance. However, this does not mean that training for doctors would not be helpful or necessary. Facilitating conditions have the second strongest positive effect on the intention to adopt BYOD. That means doctors agree that hospital management and technical infrastructure are essential requirements to adopt BYOD. Hospital management should provide technical help, support, and any support that could help BYOD adoption and foster the doctors' attitude to adopt BYOD.

In this study, social influence and response cost did not significantly correlate with adopting BYOD. Contrary to prior research of UTAUT [101, 102], social influence was not a significant factor in explaining behavioural intention. BYOD is personal, and doctors do not prefer someone else suggestions on its adoption. The result shows that doctors' decision to adopt BYOD was not influenced by peer suggestions or how they perceived it. Doctors are well educated and self-sufficient to make their own opinions. This study is consistent with other studies, especially professionals with high autonomy, like doctors [20, 86]. The findings of this study did not support the response cost hypothesis, the relationship's direction is consistent with the hypothesis, but strength is insufficient to support [52]. This study is consistent with the finding of [26, 52], which show that doctors would be willing to pay for mobile device costs at their own expense. It is probably why response costs would not negatively affect BI in the study's results. The explanation for this result may be that some doctors may have a positive view of the cost/benefit of BYOD adoption in their contexts, while others may have differing perspectives.

Perceived vulnerability results showed that the doctors' intentions were negatively affected by the perceived vulnerability and had a significant relationship with intention to adopt BYOD, but in the opposite direction than hypothesized. BYOD is personalized; doctors are already taking good care of their devices that is why some doctors may not view perceived vulnerability as a reason to reject BYOD adoption. The finding of this study shows that perceived severity significantly influences intention to adopt BYOD. Moreover, if doctors perceive a risk of adopting BYOD, their intentions are less likely to adopt BYOD, which is consistent with [63] but contrary to [26].

The adoption of BYOD was predicted by performance expectancy, effort expectancy, facilitating conditions, perceived vulnerability, perceived severity and self-efficacy. Furthermore, social influence and response cost's influence on the IAB were insignificant. Regarding the moderation effect of age and gender and after carrying out the multigroup analysis, the moderators did not significantly affect the proposed relationships. The results produced by this study have some significant contributions to the existing knowledge, methodology, and practical point of view. It provides an insightful explanation of BYOD intention factors amongst Pakistani doctors.

This study contributes to the existing literature on BYOD and provides several implications for theory. First, it provides an understanding of the predictors of BYOD adoption in general and, more specifically, within the context of Pakistan. BYOD has not been widely adopted in developing countries, and there is limited data on predictors of BYOD adoption in this region. Secondly, this study integrated the factors from the technology acceptance model UTAUT and protection motivation theory and enhanced the hybrid model's significance in the Pakistani doctors' context. Thirdly, this study expends the BYOD literature stream in the healthcare context in developing countries and Pakistan. Lastly, this study found that the hybrid model explained 87% of the variance in behavioural intention. The demographic composition of our sample of doctors in Pakistan raises questions about its generalizability to the entire population of doctors in the country. Notably, our sample exhibited a relatively small percentage of females, constituting only 15% of the sample. To assess the extent of this discrepancy, it is imperative to consider the broader doctor population in Pakistan.

The results of this study provided valuable insights for decision-makers working in the healthcare of Pakistan. This research sheds light on them by presenting a clearer picture of the real difficulties encountered by doctors' adoption of BYOD. These findings are also valuable for BYOD (mobile) system developers to design a system that meets doctors' requirements. This study may help healthcare organizations better understand doctors' perceptions of BYOD and identify strategies to engage doctors with BYOD to manage health and well-being better. This study found that performance expectancy, effort expectancy, facilitating conditions, and self-efficacy significantly impact the adoption of BYOD. Perceived vulnerability and perceived severity had been identified as a barrier to adopting BYOD, which can contribute to rejection to adopt BYOD.

Doctor's consider the use of technology and ease of use to increase the intention to adopt BYOD. Hospital's management should allocate resources to facilitate the adoption process, maintain the technologies required to operate BYOD, and enable the IT infrastructure to support it. The devices, if vulnerable to viruses or hacking, will decrease doctor's confidence in using them. IT developers need to develop protection controls that will be easy to use and practical. Doctors feel comfortable using their devices and can operate them with minimal assistance. The training program and technical support can enhance BYOD adoption; conversely, ignoring any of the factors can slow down the adoption process. The research also makes practical contributions by revealing critical challenges hospital administrators should consider when developing BYOD policies.

### Theoretical and practical contributions

This study advances existing research by integrating elements from Protection Motivation Theory (PMT)—specifically perceived vulnerability, perceived severity, response cost, and self-efficacy—into a hybrid model to enhance its validity. This integration broadens the applicability of the Unified Theory of Acceptance and Use of Technology (UTAUT) beyond traditional technology and Information Systems (IS) settings, extending it to privacy and security contexts from the perspectives of doctors and consumers. Originally, the UTAUT model explained 56% of the variance in users' intention to use technology, while its successor, UTAUT2, accounted for 74% of the variance in behavioral intention (BI). Although both models captured significant portions of BI, some of their proposed relationships may not be universally applicable across all contexts. By empirically testing the hybrid model with smart-PLS analysis, the study found that it accounts for 87% of the variance in BI. This suggests that the modified hybrid model, which revises the original UTAUT propositions, provides a more robust framework for understanding the acceptance of Information Systems and Information Technology (IS/IT) within privacy and security environments from the viewpoints of both doctors and consumers.

The study offers crucial insights for healthcare decision-makers in developing countries regarding the challenges doctors encounter when adopting Bring Your Own Device (BYOD) practices. It recommends that system developers design mobile solutions tailored to doctors' specific needs and suggests implementing awareness programs to highlight the benefits of BYOD, thereby enhancing doctors' autonomy and performance. Ensuring that BYOD systems are user-friendly is essential for boosting productivity.

Furthermore, the research underscores the importance of hospital management in supporting BYOD adoption by allocating necessary resources and strengthening IT infrastructure. Addressing security concerns, such as the vulnerability of devices to viruses and hacking, is vital to maintain doctors' trust and confidence in using their personal devices at work. By providing robust security measures and comprehensive support, organizations can facilitate the effective and secure use of BYOD in healthcare settings across developing countries.

### Limitations and future work

The study's results' generalisability is subject to some limitations that provide future researchers with the opportunity. First, the study targets online doctors who use Facebook, the situation of social turmoil forced researchers to choose the online survey method. The finding may limit the generalizability of the findings to offline or less digitally active healthcare professionals. Future research should employ stratified sampling techniques to include a broader range of healthcare professionals across diverse roles and regions, and recruit participants through professional associations to enhance sample diversity and reliability. Second, doctors are hard-to-reach populations; that is why the sample size in this study is small. The future study should have a large sample size; it might show different attitudes and behaviours towards BYOD adoption.

Third, the cross-sectional design of this study limits its ability to establish causal relationships or explore the temporal dynamics of BYOD adoption. Longitudinal studies are needed to examine how adoption behaviors evolve over time and identify causal links between influencing factors.

Fourth, this study focused exclusively on the perspectives of doctors, overlooking other stakeholders such as hospital management, IT staff, and system developers. Future research should include these perspectives to provide a more holistic understanding of BYOD adoption, exploring organizational strategies, technical challenges, and infrastructural requirement. Additionally, integrating qualitative approaches, such as interviews or focus groups, could uncover nuanced factors influencing BYOD adoption, including cultural and organizational dynamics. Lastly, while this study examined two moderators, future research should explore additional moderators or mediators, such as organizational culture, regulatory frameworks, or employee training programs, to deepen understanding of how adoption is facilitated or hindered. Mixed-methods approaches combining quantitative data on adoption rates with qualitative insights from stakeholders could further enrich the understanding of BYOD adoption dynamics. Addressing these limitations and pursuing these research directions will contribute to a more comprehensive understanding of BYOD adoption and support the development of tailored strategies to improve implementation and security practices in healthcare settings.

## Conclusions

This study aimed to identify the determinant factors that affect the adoption of BYOD among Pakistani doctors. This study extended the UTAUT model by adding the construct of PMT to develop the hybrid model of behavioural intention to adopt BYOD among Pakistani doctors. The results revealed that performance expectancy, effort expectancy, facilitating conditions, and self-efficacy positively influence intention to adopt BYOD, while perceived vulnerability and perceived severity had a negative effect on it. These results can support the healthcare organizations to understand further ways to encourage and help doctors adopting BYOD.

## Supplementary Information

Supplementary Material 1.

Supplementary Material 2.

## Data Availability

The datasets generated and analyzed during the current study are not publicly available due to confidentiality agreements with the participants. However, anonymized data may be made available from the corresponding author upon reasonable request and with permission from the ethics committee.

## Abbreviations

AVE: Average Variance Extracted
BYOD: Bring Your Own Device
CR: Composite Reliability
EE: Effort Expectancy
EHR: Electronic Health Record
FC: Facilitating Conditions
HIS: Health Information System
IAB: Intention to Adopt BYOD
IBM: International Business Management
ICT: Information and Communication Technology
LMICs: Low and Middle-Income Countries
PE: Performance Expectancy
PLS-MGA: PLS Multi-Group Analysis
PLS-SEM: Partial Least Squares Structural Equation Modelling
PMDC: Pakistan Medical and Dental Council
PMT: Protection Motivation Theory
PS: Perceived Severity
PV: Perceived Vulnerability
RC: Response Cost
SI: Social Influence
SE: Self-Efficacy
SNSs: Social Networking Sites
SPSS: Statistical Package for Social Sciences
UTAUT: Unified Theory of Acceptance and Use of Technology
