# Supplementary material for: On the perspective of doctors’ intention—a hybrid BYOD model
Source: BMC Health Serv Res. 2025 Oct 14;25:1356. doi: 10.1186/s12913-025-12801-x (PMC12519610; doi:10.1186/s12913-025-12801-x)
Supplement: Supplementary file 1 — Supplementary Material 1. [file 12913_2025_12801_MOESM1_ESM.docx]

**Codes, Descriptions, and Measurement of Variables**

| **Constructs** | **Definition** | **Codes** | **Items** | **Reference** |
| --- | --- | --- | --- | --- |
| **Performance Expectancy (PE)** | The degree to which the doctors believes that using a BYOD will help him/her to attain gains in job performance | PE1 | I expect BYOD to be useful in my job | (Venkatesh, Thong, and Xu 2012) |
|  |  | PE2 | Adoption of BYOD I expect it enables me to accomplish tasks more quickly |  |
|  |  | PE3 | Adoption of BYOD I expect it increase my productivity on the job |  |
|  |  | PE4 | Adoption of BYOD I expect significantly increase the quality of my work |  |
|  |  | PE5 | Adopting BYOD is good for my professional development |  |
| **Effort Expectancy (EE)** | The degree of ease associated with the use of BYOD | EE1 | Learning how to use BYOD is easy for me | (Venkatesh, Thong, and Xu 2012) |
|  |  | EE2 | I expect my interaction with BYOD would be clear and understandable |  |
|  |  | EE3 | I would find the BYOD easy to use |  |
|  |  | EE4 | I expect it would be easy for me to become skilful at using BYOD |  |
| **Social Influence (SI)** | The degree to which doctors perceive  that important others believe he/she  should adopt BYOD | SI1 | People who Influence my behaviour thinks that I should use the BYOD | (Venkatesh, Thong, and Xu 2012) |
|  |  | SI2 | My colleague expects that my services will be better by using the BYOD |  |
|  |  | SI3 | Hospital’s Administration has been helpful in the use of BYOD |  |
| **Facilitating Conditions (FC)** | The degree to which the doctors perceive that an organizational and technical infrastructure exists to support the use of the BYOD | FC1 | I have the resources necessary to adopt BYOD | (Venkatesh, Thong, and Xu 2012) |
|  |  | FC2 | I can get help from others when I have difficulties using BYOD service |  |
|  |  | FC3 | BYOD service is compatible with other technologies I use |  |
| **Intention to adopt BYOD (IAB)** | Refers to the expected action of the doctors regarding the actual usage of BYOD | IAB1 | I expect my interaction with BYOD would be clear and understandable | (Venkatesh, Thong, and Xu 2012) |
|  |  | IAB2 | Learning BYOD will easy for me |  |
|  |  | IAB3 | I would find the BYOD service easy to adopt |  |
| **Perceived Vulnerability (PV)** | Perceived vulnerability refers to the probability that one will experience harm | PV1 | If I will adopt BYOD, the privacy of my data will be at risk | (Sun et al. 2013) (Johnston & Warkentin, 2010) |
|  |  | PV2 | If I will adopt BYOD, my device will be at a risk of malware |  |
|  |  | PV3 | If I will adopt BYOD, malware will infect my device |  |
| **Perceived Severity (PS)** | Perceived severity refers to the degree of harm from unhealthy behaviour | PS1 | If the privacy of my device will be at risk, it be severe | (Sun et al. 2013) (Johnston & Warkentin, 2010) |
|  |  | PS2 | If my device will be at a risk of malware, it will be serious |  |
|  |  | PS3 | If malware will infect my device, it would be a significant problem |  |
| **Response Cost (RC)** | Response cost refers to any costs associated with taking the adaptive coping response. | RC1 | Employing anti-malware will require considerable investment and effort | (Sun et al. 2013) (Lee and Larsen 2009) |
|  |  | RC2 | The cost of employing anti-virus will decrease the benefits achieve from BYOD |  |
|  |  | RC3 | Employing anti-virus can slow down your device |  |
| **Self-Efficacy (SE)** | Self-efficacy is the degree to which an individual believes in his or her ability | SE1 | it will be easy for me to use BYOD | (Sun et al. 2013) (Johnston & Warkentin, 2010) |
|  |  | SE2 | I have the capability to use BYOD |  |
|  |  | SE3 | I am able to use BYOD without much effort |  |
|  |  | SE4 | I have the knowledge necessary to use BYOD |  |

**Reference**

Johnston, By Allen C, and Merrill Warkentin. 2010. “Fear Appeals and Information Security Behaviors: An Empirical Study.” *MIS Quarterly* 34 (3): 549–66.

Lee, and Kai R Larsen. 2009. “Threat or Coping Appraisal: Determinants of SMB Executives’ Decision to Adopt Anti-Malware Software.” *European Journal of Information Systems* 18 (2): 177–87. https://doi.org/10.1057/ejis.2009.11.

Sun, Yongqiang, Nan Wang, Xitong Guo, and Zeyu Peng. 2013. “Understanding the Acceptance of Mobile Health Services: A Comparison and Integration of Alternative Models.” *Journal of Electronic Commerce Research* 14 (2): 183–200.

Venkatesh, Viswanath, James Y. L. Thong, and Xin Xu. 2012. “Consumer Acceptance and Use of Information Technology : Extending the Unified Theory.” *MIS Quarterly* 36 (1): 157–78.
