# Supplementary material for: On the perspective of doctors’ intention—a hybrid BYOD model
Source: BMC Health Serv Res. 2025 Oct 14;25:1356. doi: 10.1186/s12913-025-12801-x (PMC12519610; doi:10.1186/s12913-025-12801-x)
Supplement: Supplementary file 2 — Supplementary Material 2. [file 12913_2025_12801_MOESM2_ESM.docx]

**RESEARCH INSTRUMENT**

**SECTION A: GENERAL INFORMATION**

**This section asks general questions about you**

Pleases tick as appropriate (√) the relevant box.

**1. Age:**

A) Under 25 years B) 25 – 40 years C) 41 – 56 years D) Above 56 years

**2. Gender:**

A) Male B) Female

**3. PMDC Registration No:**

**4. Medical Specialty:**

A) Family Physician B) GIM C) Pediatrics D) Surgical

E) Other

**5.** **Your Working Experience:**

A) Less than 5 years B) 5- 10 years C) 10 – 20 years D) Above 20 years

**6. Your Hospital is:**

A) Public B) Private

**7. Do you have a mobile device?**

A) Yes B) No

**7. What type of mobile device you have?**

A) Mobile Phone B) Smartphone C) Tablet D) Personal Digital Assistant (PDA)

E) Other

**8. What is the brand of your mobile device?**

A) Apple B) Samsung C) Microsoft D) Nokia

E) Q-Mobile F) Other

**9. Do you carry a mobile device while at work?**

A) Yes B) No

**10. On average in a day, how much time do you spend on your mobile device?**

A) None to 30 minutes B) 1-2 hours C) 3- 4 hours D) 5-6 hours

E) 7 hours or more

**11. On average in a day, how much of this time is your work-related?**

A) None to 30 minutes B) 1-2 hours C) 3- 4 hours D) 5-6 hours

E) 7 hours or more

**SECTION B:**

This section asks about the intention to adopt BYOD. Respondents were asked to indicate the extent to which they agreed or disagreed with each statement using 7 Likert Scale.

Please check (√) in the space provided using the following scale:

1= Strongly Disagree, 2 = Disagree, 3= Disagree Somewhat, 4= Undecided (Neutral), 5= Agree Somewhat, 6= Agree, 7= Strongly Agree

| **Performance Expectancy** | | | | | | | | |
| --- | --- | --- | --- | --- | --- | --- | --- | --- |
| **Code** | **Items** | 1 | 2 | 3 | 4 | 5 | 6 | 7 |
| PE1 | I expect BYOD to be useful in my job |  |  |  |  |  |  |  |
| PE2 | Adoption of BYOD I expect it enables me to accomplish tasks more quickly |  |  |  |  |  |  |  |
| PE3 | Adoption of BYOD I expect it increase my productivity on the job |  |  |  |  |  |  |  |
| PE4 | Adoption of BYOD I expect significantly increase the quality of my work |  |  |  |  |  |  |  |
| PE5 | Adopting BYOD is good for my professional development |  |  |  |  |  |  |  |
| **Effort Expectancy** | | | | | | | | |
| **Code** | **Items** |  |  |  |  |  |  |  |
| EE1 | Learning how to use BYOD is easy for me | 1 | 2 | 3 | 4 | 5 | 6 | 7 |
| EE2 | I expect my interaction with BYOD would be clear and understandable |  |  |  |  |  |  |  |
| EE3 | I would find the BYOD easy to use |  |  |  |  |  |  |  |
| EE4 | I expect it would be easy for me to become skilful at using BYOD |  |  |  |  |  |  |  |
| **Social Influence** | | | | | | | | |
| **Code** | **Items** | 1 | 2 | 3 | 4 | 5 | 6 | 7 |
| SI1 | People who Influence my behaviour thinks that I should use the BYOD |  |  |  |  |  |  |  |
| SI2 | My colleague expects that my services will be better by using the BYOD |  |  |  |  |  |  |  |
| SI3 | Hospital’s Administration has been helpful in the use of BYOD |  |  |  |  |  |  |  |
| **Facilitating Conditions** | | | | | | | | |
| **Code** | **Items** | 1 | 2 | 3 | 4 | 5 | 6 | 7 |
| FC1 | I have the resources necessary to adopt BYOD |  |  |  |  |  |  |  |
| FC2 | I can get help from others when I have difficulties using BYOD service |  |  |  |  |  |  |  |
| FC3 | BYOD service is compatible with other technologies I use |  |  |  |  |  |  |  |
| **Intention to Adopt BYOD** | | | | | | | | |
| **Code** | **Items** | 1 | 2 | 3 | 4 | 5 | 6 | 7 |
| IAB1 | I expect my interaction with BYOD would be clear and understandable |  |  |  |  |  |  |  |
| IAB2 | Learning BYOD will easy for me |  |  |  |  |  |  |  |
| IAB3 | I would find the BYOD service easy to adopt |  |  |  |  |  |  |  |
| **Price Value** | | | | | | | | |
| **Code** | **Items** | 1 | 2 | 3 | 4 | 5 | 6 | 7 |
| PVal1 | BYOD service is reasonably priced |  |  |  |  |  |  |  |
| PVal2 | BYOD service will be a good value for the money |  |  |  |  |  |  |  |
| PVal3 | At the current price, BYOD service provides a good value |  |  |  |  |  |  |  |
| **Perceived Vulnerability** | | | | | | | | |
| **Code** | **Items** | 1 | 2 | 3 | 4 | 5 | 6 | 7 |
| PV1 | If I will adopt BYOD, the privacy of my data will be at risk |  |  |  |  |  |  |  |
| PV2 | If I will adopt BYOD, my device will be at a risk of malware |  |  |  |  |  |  |  |
| PV3 | If I will adopt BYOD, malware will infect my device |  |  |  |  |  |  |  |
| **Perceived Severity** | | | | | | | | |
| **Code** | **Items** | 1 | 2 | 3 | 4 | 5 | 6 | 7 |
| PS1 | If the privacy of my device will be at risk, it be severe |  |  |  |  |  |  |  |
| PS2 | If my device will be at a risk of malware, it will be serious |  |  |  |  |  |  |  |
| PS3 | If malware will infect my device, it would be a significant problem |  |  |  |  |  |  |  |
| **Response Cost** | | | | | | | | |
| **Code** | **Items** | 1 | 2 | 3 | 4 | 5 | 6 | 7 |
| RC1 | Employing anti-malware will require considerable investment and effort |  |  |  |  |  |  |  |
| RC2 | The cost of employing anti-virus will decrease the benefits achieve from BYOD |  |  |  |  |  |  |  |
| RC3 | Employing anti-virus can slow down your device |  |  |  |  |  |  |  |
| **Self-Efficacy** | | | | | | | | |
| **Code** | **Items** | 1 | 2 | 3 | 4 | 5 | 6 | 7 |
| SE1 | it will be easy for me to use BYOD |  |  |  |  |  |  |  |
| SE2 | I have the capability to use BYOD |  |  |  |  |  |  |  |
| SE3 | I am able to use BYOD without much effort |  |  |  |  |  |  |  |
| SE4 | I have the knowledge necessary to use BYOD |  |  |  |  |  |  |  |
